# Household Tobacco Smoke and Admission Weight Predict Severe Bronchiolitis in Infants Independent of Deprivation: Prospective Cohort Study

**DOI:** 10.1371/journal.pone.0022425

**Published:** 2011-07-19

**Authors:** Malcolm G. Semple, David C. Taylor-Robinson, Steven Lane, Rosalind L. Smyth

**Affiliations:** 1 Department of Women's and Children's Health, University of Liverpool, Liverpool, United Kingdom; 2 Department of Public Health and Policy, University of Liverpool, Liverpool, United Kingdom; 3 Department of Biostatistics, University of Liverpool, Liverpool, United Kingdom; Murdoch Childrens Research Institute, Australia

## Abstract

**Objectives:**

To examine demographic, environmental and clinical factors associated with severe bronchiolitis in infants admitted to hospital and quantify the independent effects of these factors.

**Design:**

Prospective cohort study.

**Setting:**

Alder Hey Children's Hospital, Liverpool, United Kingdom.

**Participants:**

378 infants admitted to hospital with a diagnosis of bronchiolitis, of whom 299 (79%) were antigen positive to respiratory syncytial virus (RSV).

**Outcome:**

Severity of disease during admission, defined as “no need for supplemental oxygen” (reference group), “any need for supplemental oxygen” and “any need for mechanical ventilation”.

**Results:**

Univariate analysis found male sex (p = 0.035) and tobacco smoking by a household member (p<0.001) were associated with need for both supplemental oxygen and mechanical ventilation. Premature birth, low gestation, low birth weight, low admission weight and low corrected age on admission were also associated with need for mechanical ventilation (all p≤0.002). Deprivation scores (IMD 2004) were significantly higher in households where a member smoked compared to non-smoking households (p<0.001). The odds of smoking predicted by deprivation were 7 times higher (95%CI (3.59, 14.03)), when comparing the least and most deprived quintiles of the study population. Family history of atopic disease and deprivation score were not associated with severe disease. Multivariate multinomial logistic regression which initially included all covariates, found household tobacco smoking (adjusted OR = 2.45, 95%CI (1.60, 3.74) predicted need for oxygen supplementation. Household tobacco smoking (adjusted OR = 5.49, (2.78, 10.83)) and weight (kg) on admission (adjusted OR = 0.51, (0.40, 0.65)) were both significant predictors in the final model for mechanical ventilation. The same associations and similar size of effects were found when only children with proven RSV infection were included in analysis.

**Conclusions:**

Low admission weight and householder tobacco smoking increased the risk of severe bronchiolitis in infants admitted to hospital. These effects were independent of a standard deprivation measure. NIHR Study Ref. DHCS/G121/10.

## Introduction

Human respiratory syncytial virus (RSV) is the most common cause of acute lower respiratory tract infection in infants throughout the world.[1] There is a spectrum of disease from mild coryza to respiratory failure requiring mechanical ventilation. Bronchiolitis caused by RSV infection results in the hospital admission of between 1 and 2.5% of all infants in industrialised countries making this virus the single most common cause for hospitalisation in the first year of life.[2] Infants with severe disease requiring admission for oxygen or mechanical ventilation place a high burden upon health care resources.

Tobacco smoke is the most common and important indoor environmental pollutant to which young children are exposed. The epidemiological association between parental smoking and acute lower respiratory illness (ALRI) in children is recognized. However it has proved challenging to disentangle the direct effect of parental smoking,[3], [4] particularly as social deprivation may confound or mediate the relationship between tobacco smoke exposure and ALRI.[5] The contribution of these factors upon RSV disease is even less clear.[6]

In this study we investigated the association between environmental, demographic and clinical factors, and the severity of bronchiolitis, in infants admitted to hospital in a deprived city in the UK. Infants were prospectively recruited on admission and severe disease (any need for oxygen or mechanical ventilation) assessed after the hospital episode. Univariate and multivariate analyses were used to explore associations between the environmental, demographic and clinical factors and the need for oxygen or mechanical ventilation.

## Methods

### Study Design

Prospective cohort study.

### Setting

Alder Hey Children's Hospital, Liverpool, UK. A large urban children's hospital providing emergency, general, specialist and intensive care to some of the most socially deprived areas in England.

### Participants

#### Inclusion criteria

Infants admitted to Alder Hey Children's Hospital, Liverpool from the local community (postcodes L1 to L38) with a diagnosis of bronchiolitis were recruited over three consecutive endemic winter seasons (2002/3 & 2003/4 November through February, 2004/5 October through January). Paediatricians diagnosed bronchiolitis when infants (children<2 years of age) presented with tachypnea (>50 breaths/min), subcostal recession, and bilateral inspiratory crackles on auscultation. Admission criteria typically involved feeding difficulties, hypoxia in air, respiratory distress or respiratory failure. Infants were prospectively recruited by a single investigator (MGS) usually within 24 hours of admission. Consecutive recruitment was attempted.

#### Exclusion criteria

Infants both admitted and discharged on Saturdays and Sundays were not recruited due to restrictions on staff.

Infants with previously diagnosed haemodynamically significant congenital heart disease (cyanotic, single ventricle physiology, or acyanotic disease requiring medical therapy) were excluded on the grounds of selection and allocation bias (detailed below). The potential of use of prophylactic palivizumab (a recombinant monoclonal anti-RSV immunoglobulin) in this group to confound analysis also had bearing on the decision to exclude these infants from the study.[7]

We excluded infants living outside Liverpool, since children in these areas with mild disease are treated in sub regional hospitals, whereas children with more severe disease are selectively transferred to Alder Hey Children's Hospital.

### Variables

#### Outcome

Outcome was defined by membership of disease severity group; “no need for supplemental oxygen”, “any need for supplemental oxygen” or “any need for mechanical ventilation” during the hospital episode.[8]

### Covariates

Weight was measured on admission (kg). Household postcode was noted from the usual home address given by parents or carers on admission. Other data were collected from parents or carers at recruitment using a structured history-form. This included queries for; sex, birth weight (kg or lb & oz converted to kg), gestation (weeks), family history of atopy (defined as any first degree relative on treatment for doctor-diagnosed asthma, hay-fever or eczema), duration of illness prior to admission (days), and “current smoking by any member of the household regardless of location of where they smoked” (yes/no). When required, the investigator prompted with the 1981 UK Census top level definition of household membership “people who have the address as their only or main residence and who either share one meal a day or share the living accommodation.”[9] Prematurity was defined as birth before completing 37 weeks gestation.

Infants were managed using a standardized care pathway derived from evidence based guidelines.[10] Criteria for admission included reduced oral intake (<75% of normal) and or peripheral oxygen saturation (SpO_2_)<93% in air. Oxygen was administered to maintain SpO_2_>92%. Infants with respiratory failure and or apnoea were mechanically ventilated. Non-invasive ventilation (nasal continuous positive airway pressure) was not used during the period of this study.

Human respiratory syncytial virus (RSV) status was established in all cases by a rapid antigen ELISA (DB Directigen RSV™; Becton Dickinson UK) of nasopharyngeal aspirate (NPA) sampled on admission.

The English Index of Multiple Deprivations 2004 (IMD 2004) score was derived for each infant on the basis of his or her household postcode.[11] The IMD is a small area measure of socioeconomic status that is commonly used in Government statistics and epidemiological studies in England. The scores are available for 32,482 small geographical areas (lower super output areas, LSOAs) in England, each containing around 400 households and 1000 individuals. The IMD is composite measure of social deprivation based on seven domains; income, employment, health, education, housing, living environment and crime. (http://webarchive.nationalarchives.gov.uk/+/http://www.communities.gov.uk/archived/general-content/communities/indicesofdeprivation/216309/)

Birth to one-year-old sex ratio data for England was derived from the 2001 census (http://casweb.mimas.ac.uk).

Hospital episode summary (HES) data for all episodes that included a diagnosis of bronchiolitis in infants during the study periods provided an estimate of the total number of infants available for recruitment.

### Reduction of bias

Infants living beyond Liverpool who were admitted to Alder Hey Children's hospital were excluded to avoid selection bias. Sub-regional hospitals outside Liverpool have limited or no capacity for the highest level of care (mechanical ventilation). If included, infants living beyond Liverpool would be present in the most severe disease group without proportionate representation of infants living beyond Liverpool in milder disease groups. The resulting loss of population-based recruitment would prevent fair comparisons to be made.

Infants with previously diagnosed haemodynamically significant congenital heart disease form a recognised albeit heterogeneous high-risk group and during this study period many were planned to receive prophylactic palivizumab.[7] In this community these infants have direct access to the cardiology unit for specialist assessment and monitoring and are subject to RSV screening at entry to reduce nosocomial infection. This is not comparable to the admission and diagnostic processes for otherwise healthy infants, which involves assessment in primary care and or the emergency department with reference to national guidelines.[10] These differences precluded inclusion on the grounds of selection bias. Infants with haemodynamically significant congenital heart disease have different peripheral oxygen saturations and different target ranges to otherwise healthy infants and make more frequent use of supplemental oxygen both of which biases allocation to disease outcome groups used in this and similar studies.[8]

The average ages of infants in disease groups were 8 and 19 weeks old reducing likelihood of recall bias of birth data. Parents typically reported birth weight as lb & oz or kg to the nearest point value i.e. 100 g and gestation in whole weeks or as “term”. Many parents attended with the parent-held child health record “red book” which corroborated parent reported birth data against recorded birth weight measured in kg to nearest 5 g and gestation at birth, completed by the midwife attending birth.

The investigators had no role in the clinical management of the infants so did not influence the outcome measure.

### Statistics

No prior power calculation was made. However, as a rule of thumb 10 to 15 cases are required for each explanatory variable included in a multivariate logistic regression model. This implies that we can include at least 6 variables in a model predicting oxygen supplementation and 4 variables in a model predicting mechanical ventilation.

Demographic, environmental and clinical characteristics of the participants were analysed for associations across disease severity groups using Pearson Chi-square (Χ^2^) test and the Kruskal–Wallis one-way analysis of variance (Table 1).

**Table 1:** Demographic characteristics of 378 infants admitted with bronchiolitis and univariate analysis.

Demographic characteristics	No oxygen supplementation n = 86	Oxygen supplementation n = 241	Mechanical ventilation n = 51	Statistic & significance
Male sex	44, 51%	140, 58%	31, 61%	Χ^2^ = 6.71, p = 0.035
Premature birth	18, 21%	54, 23%	27, 53%	Χ^2^ = 21.3, p<0.001
Gestation (weeks)	38.3 (37.6, 39.0)	38.1 (37.7, 38.6)	35.8 (34.6, 36.9)	Kruskal-Wallis p<0.001
Birth weight (kg)	3.04 (2.89, 3.20)	3.04 (2.94, 3.15)	2.65 (2.40, 2.90)	Kruskal-Wallis p = 0.002
Admission weight (kg)	6.33 (5.86, 6.79)	6.13 (5.85, 6.41)	5.08 (3.14, 7.01)	Kruskal-Wallis p<0.001
Corrected age on admission (weeks)	19.0 (15.3, 22.3)	18.8 (16.1, 20.9)	7.6 (3.3, 11.8)	Kruskal-Wallis p<0.001
Duration of illness prior to admission (days)	4.2 (3.4, 5.0)	3.6 (3.2, 3.9)	3.1 (2.5, 3.6)	Kruskal-Wallis p = 0.080
Atopic family history	44, 52%	134, 57%	21, 45%	Χ^2^ = 2.71, p = 0.608
Household tobacco smoker (Yes/No, %)	41/37, 53%	154/64, 71%	32/6, 84%	Χ^2^ = 13.82, p<0.001
Index of multiple deprivations 2004	48.3 (43.8, 52.9)	51.2 (48.7, 53.6)	50.6 (44.9, 56.2)	Kruskal-Wallis p = 0.697

Nominal data expressed as number and per cent of complete data for that factor, missing data excluded. Scale data expressed as group mean and (95% Confidence Interval).† Chi-square (Χ^2^) comparison with England 2001 census birth to one year old sex data.

Variables shown to be significantly associated with the outcome when considered independently in univariate analysis can become non-significant in a multivariate model. This does not necessarily mean loss of information but implies that the non-significant variables are explaining the same or less variability in outcome as the significant variables and are therefore redundant.

Multivariate multinomial logistic regression models were constructed to identify associations between the environmental, demographic and clinical covariates and outcome measures (disease severity: any need for oxygen supplementation and any need for mechanical ventilation). The reference category for the outcome variable was “no need for oxygen supplementation”.

All covariates listed above were initially included as potential predictor variables except prematurity (a technically arbitrary dichotomous variable based on gestation (premature/term)) which was withheld in favour of gestation (a continuous variable (weeks)).

Variables were rejected in an iterative process (backwards stepwise) where the association with the outcome was not significant (p>0.1). To reduce the possible exclusion of a strong confounding variable not significantly related to outcome, all variables were also sequentially included and excluded and rejected where the OR for that variable was between 0.9 and 1.1 (10% change). Variables predicting severe disease were expressed as adjusted odds ratio (OR) with 95% confidence intervals (95%CI). Goodness of fit for each model was tested using Pearson's Chi-square and Deviance.

Separate models were constructed for all infants with bronchiolitis, and for the subgroup of infants with proven RSV infection (tables 2 and 3). A further model that included all potential covariates, regardless of significance, is provided as supplemental data (table 4).

**Table 2:** Results of multivariate analysis; independent variables predicting severe bronchiolitis in 378 infants admitted to hospital.

	Oxygen supplementation		Mechanical ventilation	
Predictor variable	Odds ratio (95%CI)	Significance (p)	Odds ratio (95%CI)	Significance (p)
Weight on admission (kg)	0.93 (0.84, 1.04)	0.194	0.51 (0.40, 0.65)	<0.001
Household tobacco smoker	2.45 (1.60, 3.74)	<0.001	5.49 (2.78, 10.83)	<0.001

The reference category is: No supplemental oxygen needed. Variables included but rejected where the Odds Ratios were not significant in both dependent outcomes included sex, gestation, birth-weight, corrected age on admission, family history of atopy, and deprivation score (IMD 2004). Goodness of fit, Pearson's Χ^2^ = 726 (p<0.001) and Deviance is not significant (p = 0.999).

**Table 3:** Results of multivariate analysis; independent variables predicting severe bronchiolitis in 299 RSV positive infants admitted to hospital with bronchiolitis.

	Oxygen supplementation		Mechanical ventilation	
Predictor variable	Odds ratio (95%CI)	Significance (p)	Odds ratio (95%CI)	Significance (p)
Weight on admission (kg)	0.94 (0.83, 1.06)	0.311	0.48 (0.36, 0.64)	<0.001
Household tobacco smoker	2.48 (1.52, 4.07)	<0.001	6.77 (3.07, 14.92)	<0.001

The reference category is: No supplemental oxygen needed. Variables included but rejected where the Odds Ratios were not significant in both dependent outcomes included sex, gestation, birth-weight, corrected age on admission, family history of atopy, and deprivation score (IMD 2004). Goodness of fit, Pearson's Χ^2^ = 663 (p<0.001) and Deviance is not significant (p = 0.999).

**Table 4:** Results of multivariate analysis including all potential covariates predicting severe bronchiolitis in 378 infants admitted to hospital.

	Oxygen supplementation		Mechanical ventilation	
Predictor variable	Odds ratio (95%CI)	Significance (p)	Odds ratio (95%CI)	Significance (p)
Gestation (weeks)	1.01 (0.94, 1.08)	0.843	0.99 (0.89, 1.11)	0.868
Birth weight (kg)	1.14 (0.68, 1.89)	0.624	0.98 (0.44, 2.20)	0.961
Sex	0.77 (0.43, 1.38)	0.374	1.28 (0.52, 3.13)	0.592
Family history of atopy	1.09 (0.61, 1.95)	0.763	0.89 (0.36, 2.21)	0.805
Index of multiple deprivations 2004	1.00 (0.99, 1.02)	0.778	1.00 (0.98, 1.03)	0.855
Corrected age on admission (weeks)	1.01 (0.97, 1.04)	0.690	1.04 (0.98, 1.11)	0.235
Weight on admission (kg)	0.86 (0.67, 1.12)	0.268	0.43 (0.26, 0.71)	0.001
Household tobacco smoker	2.23 (1.21, 4.10)	0.010	7.19 (2.28, 22.60)	0.001

The reference category is: No supplemental oxygen needed. Goodness of fit, Pearson's Χ^2^ = 609 (p = 0.040) and Deviance is not significant (p = 0.999).

Statistical analyses used PASW Statistics 18.0 software.

### Ethics

The Liverpool Children's Local Research Ethics Committee approved the study (LREC Ref 01/02/RE). Written informed consent was obtained from all parents or carers of recruited infants.

### Data sharing

The data set is not available for sharing. Confidential data handling was a requirement of ethical approval and specific assurance to this condition was given to parents and carers of infants at recruitment as part of consent. Dissemination of infant age, sex and postcode data risks deductive identification and with linked clinical and social data poses a risk of breaching patient confidentiality.

## Results

### Participants

378 infants were recruited of whom 299 (79%) were RSV positive. 3 families declined to participate. HES data for the duration of the study showed that 595 infants admitted from the local community were discharged with a final diagnosis that included bronchiolitis, of these 445 were admitted for >24 hours. Using HES data as a denominator, recruitment to this study included 64% of all admissions and 85% of admission episodes greater than 24 hours. These are conservative estimates as HES data includes diagnosis made at any time during an episode, and includes infants with haemodynamically significant congenital heart disease who were excluded from this study, whereas we only recruited infants with a diagnosis of bronchiolitis made at or shortly after admission.

### Descriptive data: Factors associated with severe disease

**Figure 1:**
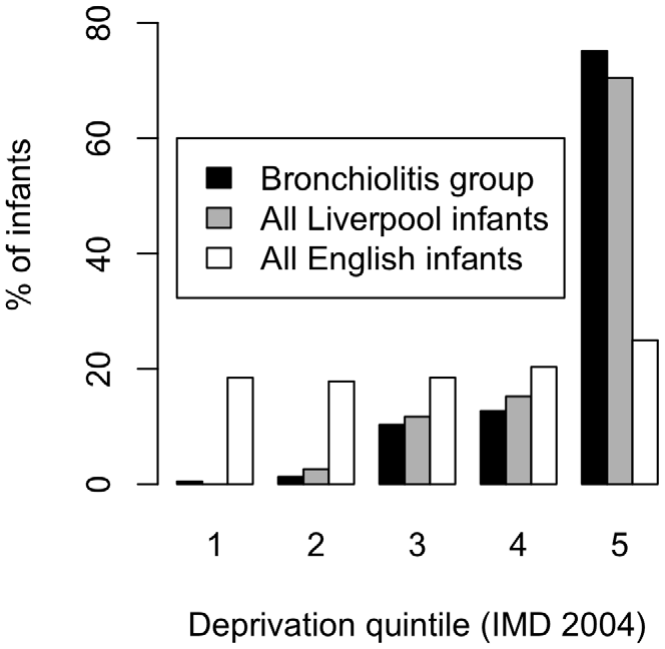
Proportion of infants by deprivation quintile. Deprivation quintiles (1 least deprived, 5 most deprived); infants recruited to this study with bronchiolitis (black), all Liverpool infants in 2001 census (grey) and all English infants in 2001 census (white).

Sex, gestation, admission weight and postcode data were complete for all cases. Birth-weight was given in 95% of cases. Household smoking behaviour was given in 91% of cases. In the 9% (33 cases) that could not answer the smoking query, most had difficulty defining household membership and a minority were reluctant to discuss smoking habits. Patient characteristics by disease severity group are summarised in table 1. Distribution of gestation and birth weight were near normal with skew to low gestation and low birth weight. Distribution of age on admission was heavily skewed to low age. Admission weight was normally distributed. Index of multiple deprivations was near normally distributed. The proportion of male infants with “no need for supplemental oxygen” (51.2%) was not significantly different from the under one year old population of England in the 2001 census (male 283,071: female 271,389, (51.1%)). Male infants were over represented in the “supplemental oxygen” and “mechanical ventilation” groups compared with the under one year old population for England (p = 0.035). Prematurity (birth before completing 37 weeks gestation), low gestation, low birth weight, low admission weight and corrected age on admission were significantly associated with need for mechanical ventilation (all p≤0.002). There was a less significant association for short duration of illness to be associated with mechanical ventilation (p = 0.080). Smoking by a household member was significantly associated with both the need for oxygen supplementation (p = 0.003) and mechanical ventilation (p<0.001). Figure 1 describes proportions of recruited infants with bronchiolitis in each deprivation quintile and compares with local (Liverpool) and national infant census data from 2001. Deprivation scores (IMD 2004) did not vary significantly between disease groups.

### Outcome data: Factors independently predicting severe disease

Multivariate multinomial analysis using logistic regression found that infants from tobacco smoking households were at increased risk of severe disease needing supplemental oxygen (OR = 2.45, 95%CI (1.60 to 3.74), p<0.001) or mechanical ventilation (adjusted OR = 5.49 (2.78 to 10.83), p<0.001) (table 2). Weight (kg) on admission reduced the risk of needing for mechanical ventilation (adjusted OR 0.51 (0.40 to 0.65), p<0.001), meaning for every 1 kg increase in admission weight, odds of an infant requiring mechanical ventilation were halved. The same predictor variables were retained when participants in the model were restricted to those with proven RSV infection and the sizes of effects were very similar (table 3). Predictor variables tested where the OR was not significant at p<0.1 in either outcome included sex, gestation, birth-weight, corrected age on admission, family history of atopy and deprivation score (IMD 2004). A less parsimonious model that included all covariates regardless of significance identified the same predictor variables with very similar sizes of effects (table 4). Inclusion of an interaction term between smoking and deprivation was not significant (data not shown).

### Other analyses

Deprivation scores were significantly higher for households where a member smoked tobacco compared to non-smoking households [IMD 2004 mean (95%CI); tobacco smoking households 55.5 (53.1 to 57.9), non-smoking households 42.0 (38.1 to 45.8), p<0.001)].

In a separate binomial logistic regression analysis, odds of smoking as outcome predicted by deprivation was 1.04 (1.02 to 1.05) per unit increase in IMD 2004, giving an adjusted OR of 7.0 (3.59 to 14.03) when comparing the least and most deprived quintiles of the study population.

Post hoc analysis of hospital records of recruited cases for prior administration of palivziumab found one case.

## Discussion

### Principal findings

Young age, low gestation, premature birth and householder smoking were risk factors associated with severe bronchiolitis requiring oxygen supplementation or mechanical ventilation when assessed using univariate analysis. This corroborates with the risk factors associated with admission to hospital published in current evidence based guidelines.[10] Male sex was significantly associated with severe disease, a finding that is frequently found in many other species when infected by virus, bacteria, fungi, and parasites.[12]

Weight on admission was strongly associated with severe disease when assessed by univariate analysis and in the multivariate model was a significant independent major effect predicting need for mechanical ventilation. Weight on admission was retained in the model in preference to gestation, birth-weight, and corrected age on admission. Low weight on admission is not generally recognized as a risk factor for severe bronchiolitis in otherwise healthy infants but has been described.[13] An infant's weight is determined by many factors with complex interactions. These will include genetic influences, gestation, corrected age, family size, mode of feeding and deprivation.[14], [15], [16] The relevance of admission weight to risk of severe bronchiolitis probably reflects the value of weight as a surrogate marker of airway calibre.

No association was found between family history of atopy and severe bronchiolitis disease. Deprivation predicted risk of tobacco smoking, but deprivation was not associated with disease severity. Tobacco smoking by a household member remained a major independent predictor of the need for both supplemental oxygen and mechanical ventilation in infants with bronchiolitis. The size of effects (OR) for weight and smoking were similar in the subgroup of 299 infants with confirmed RSV infection.

Multivariate multinomial logistic regression was chosen in preference to ordinal regression as no assumption was made that membership of the disease severity groups was ordinal. The data supports this choice, as there is a distinct membership effect observed with admission weight predicting need for mechanical ventilation but not need for supplemental oxygen. This may reflect the different criteria that are used by clinical staff when deciding to initiate oxygen therapy (usually in response to hypoxia determined by peripheral oxygen saturation monitoring) and mechanical ventilation (recognition of respiratory exhaustion and or apnoea).

The association between parental or household tobacco smoke exposure and incidence of bronchitis, bronchiolitis and other lower respiratory tract infections in infancy has recently been the subject of a systematic review and meta-analysis.[17] In contrast our study examines factors influencing *severity* of bronchiolitis in infants admitted to hospital and importantly includes a standard measure of socioeconomic status in the multivariate regression analysis.

Bradley *et al* studied home allergen exposure, family history and host factors with particularly attention to atopy for association with severe bronchiolitis, in the ‘RSV Bronchiolitis in Early Life’ prospective cohort study (St Louis, Missouri). [4] Bradley *et al* described a trend between postnatal maternal cigarette smoking and reduced peripheral oxygen saturation in exposed infants (SpO_2_% decrease 2.30% (95%CI-0.05, +4.66)). Measures of socioeconomic status were not included in their multivariate regression analysis preventing exploration of any effect of deprivation or confounding by deprivation upon outcome. Bradley *et al* also suggested that absence of a family history of atopy and particularly absence of maternal asthma reduced peripheral oxygen saturation in infants (SpO_2_% decrease 2.75% (95%CI-0.05, +5.55)). In our study family history of atopy was rejected from the multivariate models as not having any significant effect upon outcome. This may be because our study was based on family history of *any* atopic disease (asthma, hay fever or eczema) in any first degree relative rather than specifying asthma in the mother.

Two large cohort studies have used multivariate regression analysis to identify risk factors for diagnosis of bronchiolitis and incidence of RSV ALRI in the community. Neither of these studies examined factors associated with severe disease in infants admitted to hospital requiring high levels of care but it is worth contrasting their findings with ours to identify common predictor variables.

In the Tucson Children's Respiratory Study Holberg *et al* used “incidence of RSV infection in infants presenting to office based paediatricians with ALRI for assessment” as an outcome measure in a prospective cohort study of 1,179 infants.[18] Multivariate logistic regression analysis found male infants and infants who shared a bedroom with others were at increased risk of RSV ALRI but there was no increased risk with current maternal smoking. However when the outcome measure was broadened by Wright *et al* to “paediatrician diagnosed ALRI”, maternal smoking, sharing bedrooms, and use of day care all independently increased the odds of ALRI.[19]

Koehoorn *et al* retrospectively examined 93,058 maternity and linked infant's records for variables predicting 12,474 outpatient and or hospital episodes coded as bronchiolitis in the Georgia Air Basin Area (South West Canada).[20] Multivariate logistic regression analysis found excess cases of bronchiolitis occurred in infants who were male, had low birth weight, were born to young mothers, and mother that smoked during pregnancy, lived in postcode areas with low maternal post-secondary education and did not initiate breast feeding in hospital. Infant demographics, clinical data at presentation with bronchiolitis and post-natal exposure to tobacco smoke were not studied.

The “Iron Chain” that links smoking and deprivation is well described, with the prevalence of smoking being highest in the areas of greatest deprivation.[21] Smoking is an important mediator in the pathway linking low socioeconomic status in childhood to adverse health outcomes in adulthood.[22] A study conducted during the same period as ours estimated adult smoking prevalence in Liverpool to be between 42% and 52%.[21] We do not dispute others' findings that deprivation is associated with incidence of RSV infection, diagnosis of bronchiolitis and need for hospital admission, none of which were outcomes in this study. Summary IMD scores rank Liverpool as the most deprived of all 354 local authority districts in England and all 149 County Councils in England (IMD 2004 Local Authority and County Council summary data sets). In our study, deprivation was not associated with severe bronchiolitis, however deprivation predicted likelihood of smoking, and smoking predicted likelihood of severe disease. Finding that deprivation was not associated with severe disease in our study may be because few infants were admitted from the few affluent areas of Liverpool and IMD 2004 scores were similarly high for most infants admitted to hospital (figure 1). This phenomenon, which effectively controlled for deprivation in the study subjects, allowed us to show that household exposure to tobacco smoke has a strong independent effect upon risk of severe bronchiolitis. However, it remains likely that deprivation continues to be an important up-stream cause of severe disease in childhood.

### Limitations

Infants both admitted and discharged on Saturdays and Sundays were not recruited and some infants admitted on weekdays for less than 24 hours were missed. Infants both admitted and discharged at weekends and for other short periods have mild disease and may have different demographics to those admitted during weekdays. However these missed cases were a minority, and overall mild cases were otherwise well represented in the study. Recruitment included at least 64% of all admission episodes greater than 4 hours and 85% of all episodes greater than 24 hours with a final diagnosis of bronchiolitis. Thus the missed cases are thought unlikely to have introduced significant selection bias in the analysis.

This study was hospital based. Alder Hey Children's' Hospital is the main provider of acute paediatric services in Liverpool, and thus we expect to have captured most of the cases of bronchiolitis in Liverpool.

We did not have access to individual level measures of deprivations and instead used an accepted small-area based measure of deprivations. This conflates the possibly separate effects of deprivation operating at the individual and area level. It remains likely that social deprivation in the community has a major independent effect on risk of admission to hospital with bronchiolitis, but conditional upon this, there does not appear to be an effect on disease severity.[17]

The smoking question related to current behaviour by any member of the infant's household. The relative influence of paternal *vs*. maternal *vs*. both smoking was not studied, nor was maternal smoking during pregnancy. Most parents who smoke during pregnancy continue to smoke postpartum. It is difficult to identify sufficiently large subgroups of children who are exclusively exposed in utero or after birth.[23]

It is possible that that disease severity at admission may influence parental honesty in reporting smoking habits however we found no evidence for this as there was no significant difference in the distribution of missing data for smoking between disease severity groups. Also allocating all missing data to either smoking or no-smoking values did not change the factors retained in the models or significantly alter the OR of the models. The validity of self-reported smoking has been the subject of review and meta-analysis, which concluded that self-reports of smoking, particularly by adults, are mostly accurate.[24]

Our study did not enquire about breastfeeding. Holberg *et al* found less than one month of breast-feeding had an independent detrimental effect of upon incidence of RSV ALRI in infants. Koehoorn *et al* found breast-feeding initiation in the maternity hospital had an independent protective effect upon risk of subsequent hospital admission with bronchiolitis. In England deprivation predicts establishment and duration of breastfeeding.[25]

The relationship between exposure to household tobacco smoke and severe bronchiolitis is likely to be causal because a dose-response effect was observed where disease severity increased as the proportion of infants exposed to tobacco smoke increased. This corroborates with biochemical studies on infants.[26] However as this is not a randomised controlled trial we cannot prove causation. Instead we assert that smoking by a household member doubles the odds of an infant admitted with bronchiolitis needing oxygen and along with low admission weight increases five-fold the odds of needing mechanical ventilation.

### Generalizability

The findings are likely to be generalizable to similar populations in urban areas of high deprivation in industrialized nations. The findings may be generalizable to other viral respiratory tract infections in young children.

### Conclusion

There are many papers describing a strong association between household smoking and incidence of lower respiratory tract infection in infants. We add to the field by describing that householder smoking and low admission weight both have major independent effects predicting severe bronchiolitis in infants admitted to hospital. The demographics of the population studied and the method of analysis show these effects can be independent of a recognized measure of multiple deprivations.
